# Isoflavones with Multifaceted Activities Synergistically Sensitize *Pseudomonas aeruginosa* to Antibiotics In Vitro and In Vivo

**DOI:** 10.3390/antibiotics15090829

**Published:** 2026-08-26

**Authors:** Dan-Dan Li, Tong Xia, Xin-Yu Zhang, Huiyan Li, Wen-Xin Niu, Tie Yao, Joon-Hee Lee, Li-Li Wang

**Affiliations:** 1 School of Chinese Materia Medica, Tianjin University of Traditional Chinese Medicine, Tianjin 301617, China; ldd0930@tjutcm.edu.cn (D.-D.L.); xt15055969110@163.com (T.X.); 13821131526@163.com (X.-Y.Z.); 2 College of Pharmacy, Pusan National University, Busan 46241, Republic of Korea; lihy0522@naver.com (H.L.); niuwenxin@naver.com (W.-X.N.); 3 School of Pharmaceutical Sciences, Liaoning University, Shenyang 110036, China; tieyao1215@163.com; 4 Research Institute for Drug Development, Pusan National University, Busan 46241, Republic of Korea

**Keywords:** *Pseudomonas aeruginosa*, anti-pathogenic, daidzein, genistein, quorum sensing, antibiotic susceptibility

## Abstract

**Background/Objectives**: *Pseudomonas aeruginosa* is a notorious multidrug-resistant pathogen that causes serious acute and chronic infections by employing quorum sensing (QS)-regulated virulence, biofilm formation, and host-damaging inflammation. To overcome the yield limitation of two previously identified marine secondary metabolites with dual QS inhibitory and PPAR-γ agonistic activities, we further screened marine-derived natural products for more abundant candidates with similar anti-virulence and anti-inflammatory properties. **Methods**: In this study, isoflavones were evaluated for anti-QS and PPAR-γ transactivation activities using reporter gene assays, and for antibacterial, anti-virulence, and anti-inflammatory effects via broth microdilution, biofilm, *G. mellonella* infection, and ELISA cytokine assays. **Results**: Daidzein and genistein were selected for their optimal anti-QS and PPAR-γ activation activities. They inhibited a key QS regulator and suppressed pyocyanin production and biofilm formation in *P. aeruginosa* without affecting bacterial growth, indicating minimal selective pressure for resistance. In addition, daidzein and genistein were found to synergistically sensitize the wild-type *P. aeruginosa* strain to gentamicin, carbenicillin, tobramycin, ampicillin, and polymyxin B, and synergistically or partially synergistically sensitize a multidrug-resistant strain to gentamicin, tobramycin, and ampicillin. Moreover, a *Galleria mellonella* infection model confirmed that daidzein and genistein significantly enhance the efficacy of gentamicin against *P. aeruginosa* infection in vivo. Furthermore, in host macrophages, daidzein and genistein significantly inhibited LPS-induced production of NO, IL-6, and IL-1β when combined with an RXR agonist, implying a protective effect on host tissues through PPAR-γ activation. **Conclusions**: These findings demonstrate that daidzein and genistein serve as effective adjuncts to conventional antibiotics, exerting multifaceted actions against *P. aeruginosa* infection.

## 1. Introduction

*Pseudomonas aeruginosa*, a Gram-negative opportunistic pathogen, is notable for its production of diverse virulence factors, robust biofilm formation, and induction of excessive inflammation in the host, all of which contribute to its pathogenesis in infections such as burn wounds, cystic fibrosis, and immunocompromised states [1,2,3]. Currently, antibiotic therapy remains the mainstay for the management of *P. aeruginosa* infections in the clinic. However, the presence of both intrinsic and acquired resistance mechanisms enables *P. aeruginosa* to rapidly develop resistance to most antibiotics [4,5]. Novel antimicrobial agent research, antibiotic alternative screening, and efficacy prolongation of existing antibiotics are the most promising strategies to combat antibiotic resistance [6]. However, even after substantial time and resources are invested in developing a new antibiotic with a novel target, it will face resistance within a short period after its introduction into clinical use [7,8]. Moreover, despite extensive research into antibiotic alternatives, very few viable options can completely replace antibiotics [6]. Therefore, rather than solely focusing on new discoveries, exploring innovative strategies to extend the useful life of existing antibiotics represents a promising path forward.

Quorum sensing (QS) is a cell-density-dependent communication mechanism that controls the transcription of approximately 10% of all *P. aeruginosa* genes, including those involved in virulence factors, biofilm formation, and antibiotic susceptibility [9]. Quorum sensing inhibitors (QSIs) have been demonstrated to attenuate bacterial pathogenicity through disrupting three interlinked QS systems (las, rhl, and pqs) but do not suppress bacterial growth. It is believed that QSIs exhibit anti-pathogenicity activity with a low likelihood of inducing antibiotic resistance [10] and might be antibiotic sensitizers. Significantly, in recent years, increasing evidence has indicated that inhibition of the QS system can synergize with antibiotics such as ciprofloxacin, ceftazidime, and gentamicin [11,12,13,14]. Therefore, QSIs represent a promising alternative or potent adjunct to traditional antibiotics for multidrug-resistant infections [10].

Despite virulence factor production and biofilm formation, QS genes or QS autoinducers (such as 3OC12-HSL) in *P. aeruginosa* were confirmed to inhibit the expression of peroxisome proliferator-activated receptor γ (PPAR-γ) in the host cells [15]. PPAR-γ, a nuclear receptor of the PPAR family (isoforms α, δ/β, and γ), heterodimerizes with retinoid X receptor (RXR) upon ligand activation; this complex binds to PPAR response elements (PPREs) to initiate transcription of target genes involved in lipid and glucose metabolism, inflammation, and cancer [16]. When *P. aeruginosa* infects the host cell, a strong inflammatory response would be quickly triggered through the production of virulence factors such as lipopolysaccharides (LPS) and exotoxin A, among others [3,17,18,19]. However, upregulation of PPAR-γ expression could enable the host to combat *P. aeruginosa* infection and attenuate the excessive inflammatory response [20,21]. Therefore, targeting PPAR-γ in host cells represents a potential strategy to combat *P. aeruginosa* infection.

In our previous study, two natural products from a marine-derived fungus were identified as PPAR-γ activators with anti-QS activity and were further proposed as promising anti-virulence, anti-biofilm, and immunostimulating agents against *P. aeruginosa* via PPAR-γ activation [22]. However, these two compounds are produced in limited quantities, which prompted us to screen a broader range of marine-derived natural products for more accessible candidates with comparable anti-virulence and anti-inflammatory efficacy. Accordingly, daidzein and genistein, isolated from the jellyfish-derived fungus *Cladosporium oxysporum*, were selected based on their optimal anti-LasR and PPAR-γ activation activities [23]. Importantly, compared with our previously identified compounds, daidzein and genistein are not only more readily accessible from natural sources but also commonly present in dietary products, which renders them more favorable for future translational development. They were found to sensitize wild-type and multidrug-resistant *P. aeruginosa* strains to antibiotics in vitro and in vivo. Furthermore, daidzein and genistein were shown to inhibit LPS-induced inflammatory response when combined with an RXR agonist via PPAR-γ activation. These findings suggest that daidzein and genistein serve as effective anti-pathogenic agents and adjuncts to antibiotics, exerting multifaceted (bacteria-directed and host-directed) actions against *P. aeruginosa* infection.

## 2. Results

### 2.1. Daidzein and Genistein Exhibited Anti-QS and PPAR-γ Transactivation Activities

**Figure 1:**
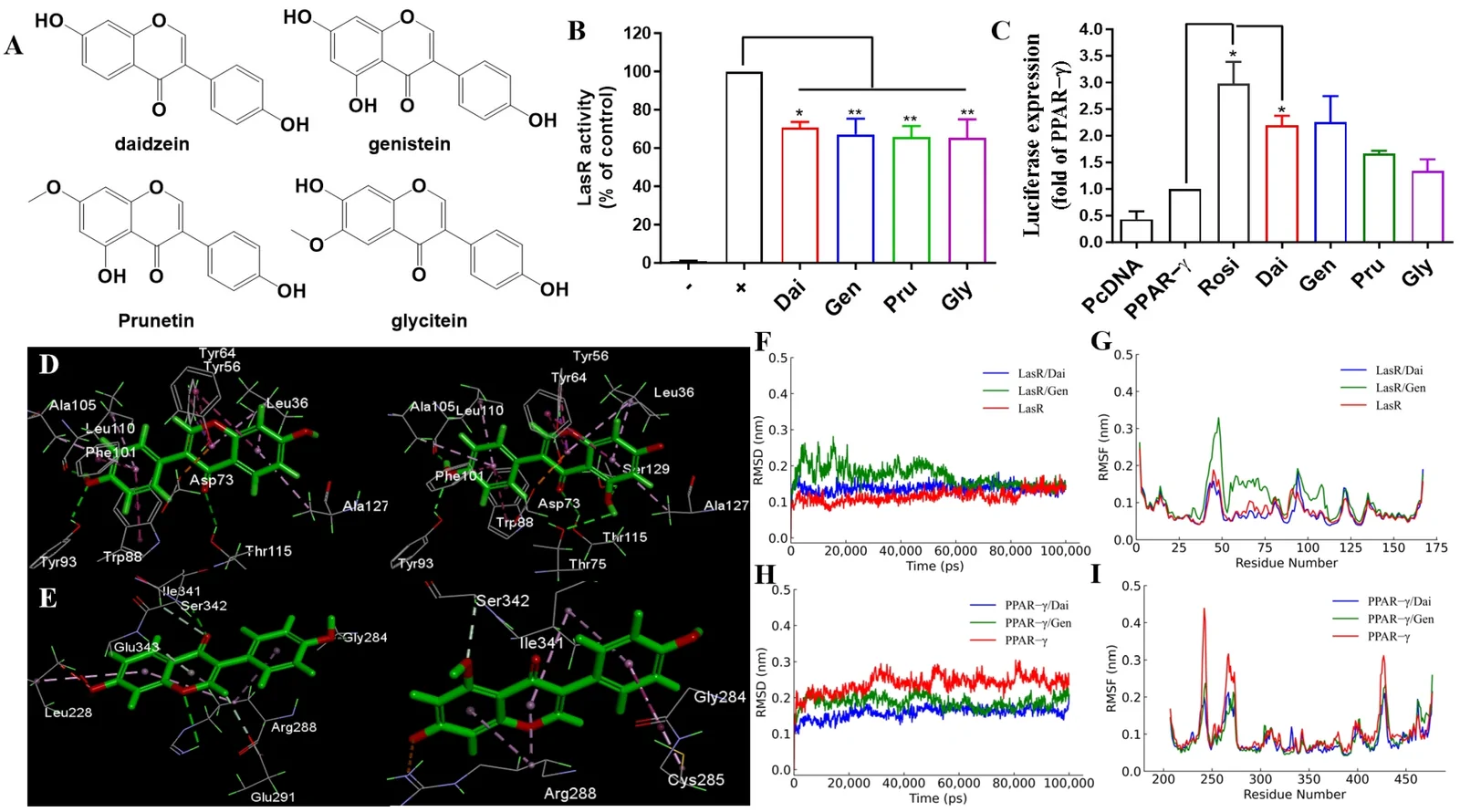
Daidzein and genistein inhibit QS response of *P. aeruginosa* and transactivate PPAR-γ. (**A**) Chemical structures of the four isoflavones evaluated in this study: daidzein, genistein, prunetin and glycitein. (**B**) Anti-LasR activities of the four isoflavones were evaluated at 10 μM using an *E. coli* dual-plasmid reporter system. Briefly, *E. coli* was co-transformed with the LasR-expressing plasmid (pJN105L) and the cognate reporter plasmid carrying a *lasI* promoter-*lacZ* fusion. β-galactosidase activity was then measured to assess the inhibitory effects of the isoflavones on LasR. (**C**) PPAR-γ transactivation effects of the four isoflavones were assessed at 10 μM by luciferase reporter assay in Ac2F cells. In this system, cells co-transfected with a PPAR-γ expression plasmid and a PPRE-luciferase reporter plasmid served as the control, whereas cells transfected with an empty PcDNA vector and PPRE-luciferase plasmid were used as the blank control. (**D**,**E**) Molecular docking analysis of the binding interactions between daidzein and genistein with LasR (**C**) and PPAR-γ (**D**). Daidzein and genistein are shown in green; hydrogen bonds are represented as green dashed lines; hydrophobic interactions are shown as pink dashed lines. (**F**–**I**) Root-mean-square deviation (RMSD) (**F**,**H**) and root-mean-square fluctuation (RMSF) (**G**,**I**) analyses of LasR (**F**,**G**) and PPAR-γ (**H**,**I**) complexes during 100 ns molecular dynamics simulations. *, *p* < 0.05 and **, *p* < 0.01.

Four isoflavones (daidzein, genistein, prunetin and glycitein; Figure 1A) isolated from the jellyfish-derived fungus *Cladosporium oxysporum* [23] were screened for anti-QS activity and PPAR transactivation effects using β-galactosidase reporter assay and Luciferase reporter assay. To experimentally validate that these four isoflavones act as antagonists of the QS regulator, a LasR-expressing plasmid (pJN105L) was co-transformed into *E. coli* along with its cognate reporter plasmid (*lasIp-lacZ* fusion plasmid). Competition between the QS signal 3OC12-HSL and the test four isoflavones was then evaluated. As shown in Figure 1B, all isoflavones significantly inhibited LasR activity at 10 μM. However, when the PPRE-x3-TK-luciferase reporter plasmid and the full-length human PPAR-γ1 expression vector were co-transfected into the Ac2F cells, PPAR-γ transactivation activity was only stimulated by daidzein and genistein, in comparison with the positive PPAR-γ agonist rosiglitazone (Figure 1C). Therefore, taken together, daidzein and genistein were chosen for subsequent experiments.

Furthermore, molecular docking simulation was performed to evaluate the interactions of daidzein and genistein with LasR and PPAR-γ. As shown in Figure S1 and Table S1, daidzein and genistein occupied the same ligand-binding domain of LasR but exhibited lower LibDockScore than the native ligand (3OC12) (Table S1), which is consistent with the results of the β-galactosidase reporter assay (Figure 1B). In addition, daidzein interacts with LasR by forming hydrogen bonds with Tyr^93^, Thr^115^, and Leu^110^, while genistein forms hydrogen bonds with LasR at residues Tyr^93^, Thr^115^, Leu^110^, Thr^75^, and Ser^129^ (Figure 1D and Table S1). Regarding PPAR-γ, daidzein forms hydrogen bonds with Ser^342^, Ile^341^, Glu^343^, Arg^288^, Glu^291^, and Gly^284^, whereas genistein forms a hydrogen bond with Ser^342^ (Figure 1E and Table S1). It has been reported that full agonists of PPAR-γ stabilize helix 12 (H12) through interaction with Tyr^473^ via a hydrogen bond (Figure S1), a key feature that distinguishes them from partial agonists, which typically occupy regions near H3 and the β-sheet [23,24,25]. This suggests that daidzein and genistein may act as partial agonists of PPAR-γ, suggesting that they may evade the weight gain and obesity-related side effects associated with full agonists [23,26].

Furthermore, to evaluate the stability of the docked complexes of daidzein and genistein with LasR and PPAR-γ, molecular dynamics (MD) simulations by GROMACS were conducted. The results clearly show that the protein-form, daidzein–protein and genistein–protein, complexes exhibit low RMSD (Figure 1F,H). Notably, when comparing the protein RMSF values with the complex protein RMSF, the daidzein–protein and genistein–protein complexes showed comparable or low deviations (Figure 1G,I). The radius of gyration (Rg) remained nearly constant during the entire simulation (Figure S1E,F), indicating that both LasR and PPAR-γ retained compact and structurally stable conformations after isoflavone binding. Collectively, these results demonstrate that daidzein and genistein can form stable complexes with LasR and PPAR-γ, supporting their potential roles as QS modulators and PPAR-γ agonists.

### 2.2. Daidzein and Genistein Inhibited Pyocyanin Generation and Biofilm Formation of P. aeruginosa Through Disrupting QS

**Figure 2:**
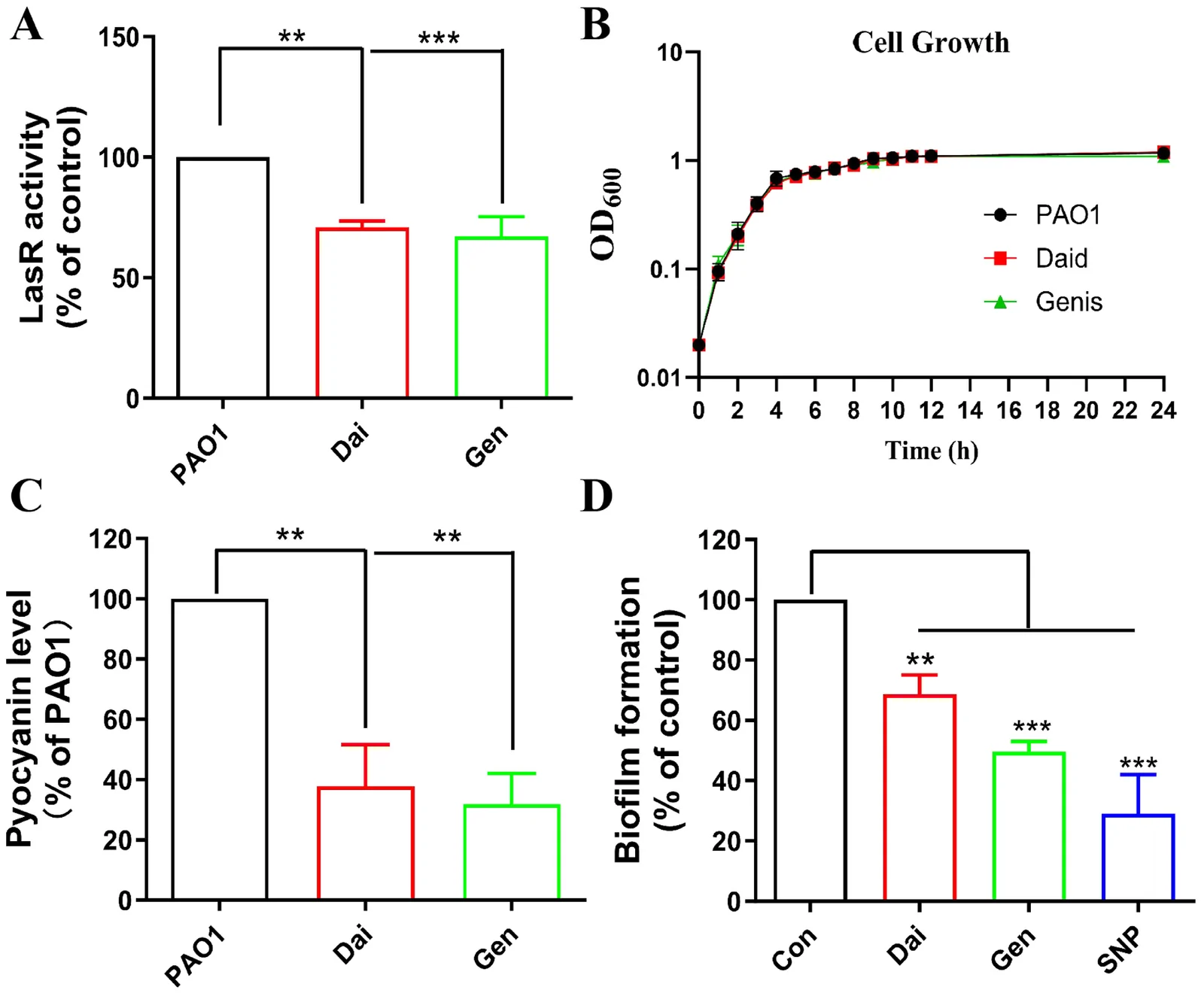
Daidzein and genistein inhibit pyocyanin production and biofilm formation in *P. aeruginosa*. (**A**) Anti-LasR activities of daidzein and genistein (10 μM) were evaluated using a *P. aeruginosa* reporter system (PAO1 carrying a lasI promoter-lacZ fusion). β-galactosidase activity was then measured to assess the inhibitory effects of daidzein and genistein on LasR. (**B**) Growth curves of *P. aeruginosa* PAO1 treated with 100 μM daidzein and genistein over 24 h. Bacterial growth was monitored by measuring OD_600_ at 2 h intervals. No significant inhibition of bacterial growth was observed at the tested concentration. (**C**) Pyocyanin production in *P. aeruginosa* PAO1 was quantified after 24 h of treatment with daidzein and genistein at 1 μM. Data are presented as percentages relative to the untreated control (set as 100%). (**D**) Anti-biofilm activity of daidzein and genistein (1 μM) was assessed using a static biofilm formation assay in 96-well plates. Sodium nitroprusside (SNP, 5 μM) was used as the positive control. **, *p* < 0.01 and ***, *p* < 0.001.

Prior to evaluating the anti-QS, anti-biofilm, and anti-virulence activities of daidzein and genistein against *P. aeruginosa*, their minimal inhibitory concentration (MIC) values were measured. In contrast to the low MICs of gentamicin, the MICs of both daidzein and genistein against *P. aeruginosa* PAO1 and multidrug-resistant MRPA strains exceeded 10 mM. Therefore, low (sub-MIC) concentrations of these compounds were used in subsequent experiments. To confirm the anti-QS activity of daidzein and genistein in *P. aeruginosa*, a reporter strain was constructed by transforming the pSC11 plasmid into PAO1 competent cells. The results further confirmed that daidzein and genistein potently suppressed LasR activity at 10 μM (Figure 2A). Meanwhile, we found that daidzein and genistein did not affect the cell growth of *P. aeruginosa* at 100 μM (Figure 2B). These results imply that daidzein and genistein exert less selective pressure on *P. aeruginosa* growth, which is consistent with a lower propensity to induce resistance [27].

Pyocyanin, a specific virulence factor tightly controlled by the QS system, is toxic to host cells [28]. Consequently, it serves both as a diagnostic marker and as a promising target for anti-virulence therapy. Daidzein and genistein significantly suppressed the production of pyocyanin (Figure 2C). In addition, the development of biofilm formation regulated by the QS system was demonstrated to be closely correlated with the pyocyanin concentration of *P. aeruginosa* [29]. Therefore, the biofilm formation assay was performed in this study. The results showed that daidzein and genistein significantly inhibited the biofilm formation of *P. aeruginosa* (Figure 2D).

### 2.3. Daidzein and Genistein Synergistically Enhance the Efficacy of Antibiotics Against P. aeruginosa In Vitro and In Vivo

**Figure 3:**
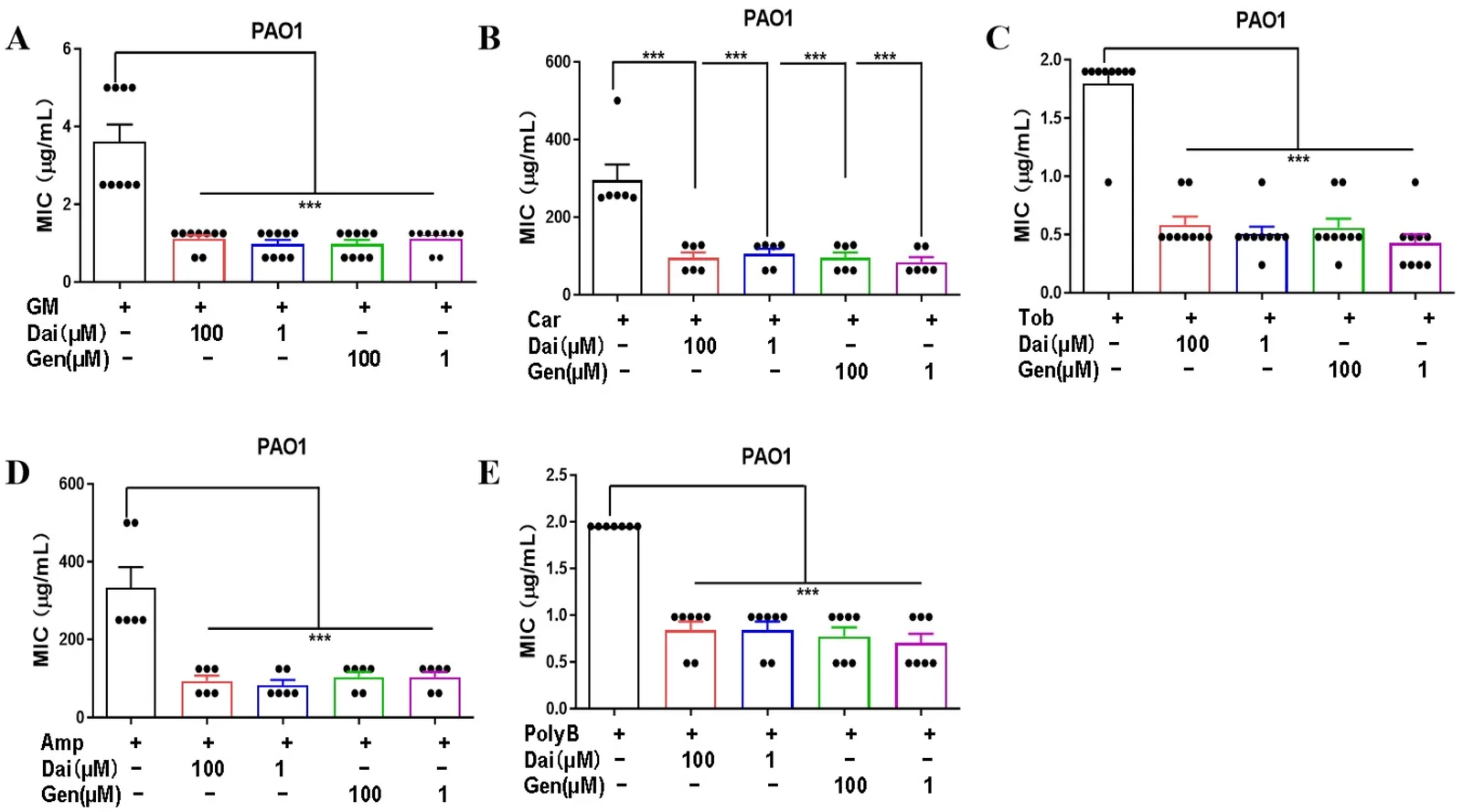
Minimum inhibitory concentration (MIC) of antibiotics combined with daidzein and genistein against *P. aeruginosa* (PAO1). MIC values of (**A**) gentamicin (GM), (**B**) carbenicillin (Car), (**C**) tobramycin (Tob), (**D**) ampicillin (Amp), and (**E**) polymyxin B (Poly B) were determined alone or in combination with daidzein (Dai) and genistein (Gen) at subinhibitory concentrations (1 and 100 μM) against *P. aeruginosa* PAO1 using the broth microdilution method in 96-well plates. DMSO (≤0.2%, *v*/*v*) was used as the solvent control. The dots represent the MIC values obtained from each individual experiment. Data are representative of at least three independent experiments. ***, *p* < 0.001.

**Figure 4:**
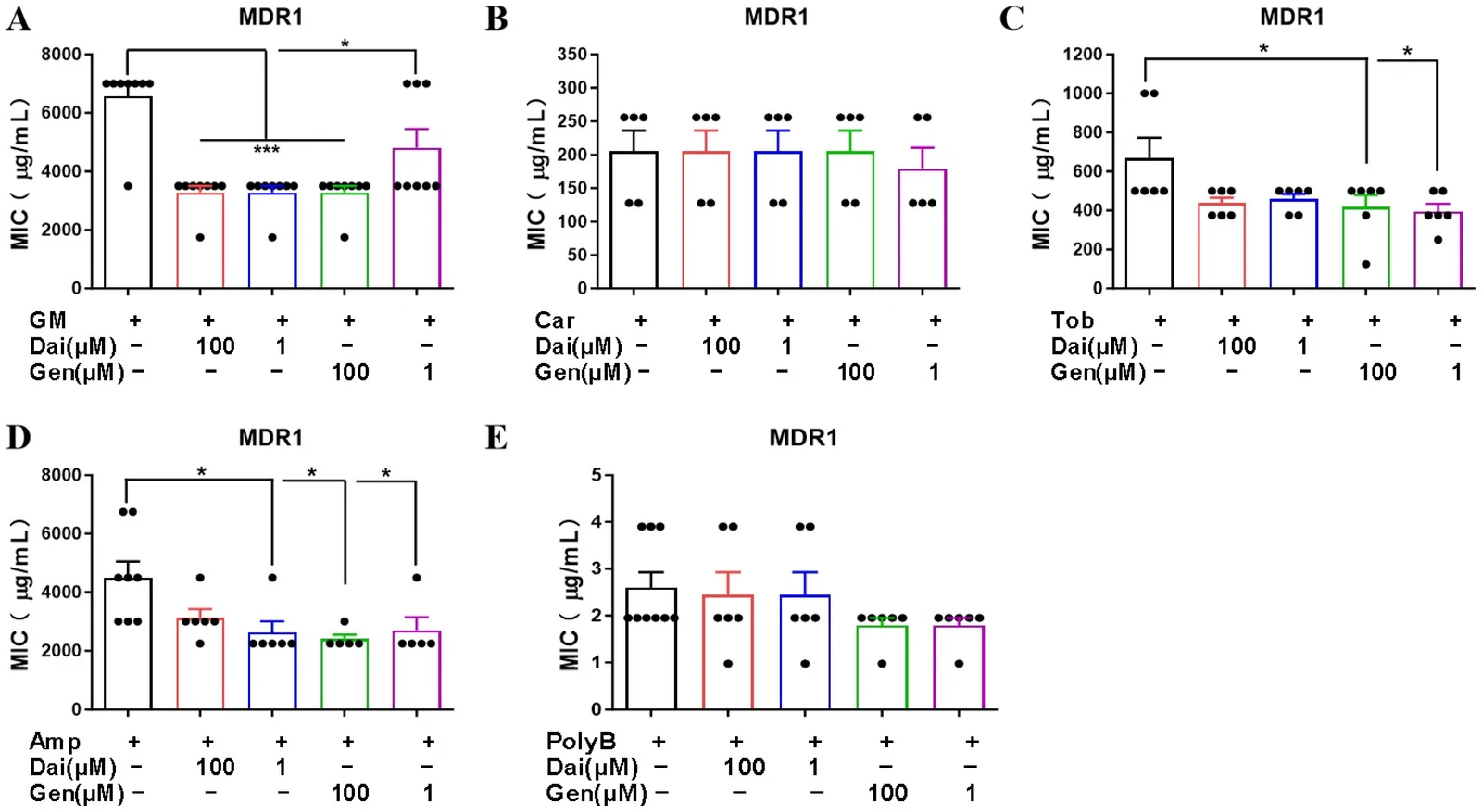
Minimum inhibitory concentration (MIC) of antibiotics combined with daidzein and genistein against *P. aeruginosa* (MRPA). MIC values of (**A**) gentamicin (GM), (**B**) carbenicillin (Car), (**C**) tobramycin (Tob), (**D**) ampicillin (Amp), and (**E**) polymyxin B (Poly B) were determined alone or in combination with daidzein (Dai) and genistein (Gen) at subinhibitory concentrations (1 and 100 μM) against MRPA using the broth microdilution method in 96-well plates. DMSO (≤0.2%, *v*/*v*) was used as the solvent control. The dots represent the MIC values obtained from each individual experiment. Data are representative of at least three independent experiments. *, *p* < 0.05 and ***, *p* < 0.001.

Since daidzein and genistein significantly inhibited pyocyanin and biofilm formation of *P. aeruginosa*, we explored whether the antibiotics can be reduced in their presence. The synergistic effect of combining daidzein/genistein with gentamicin (GM), carbenicillin (Car), tobramycin (Tob), ampicillin (Amp), and polymyxin B (Poly B) was evaluated against both PAO1 and MRPA. The MIC values are presented as the lowest concentration of each antibiotic that completely inhibited visible bacterial growth after 16 h of incubation at 37 °C. As shown in Figure 3, both isoflavones significantly enhanced the antibacterial activity of all five antibiotics against PAO1. The results of FICI calculations confirmed the synergistic interaction between daidzein/genistein and all antibiotics (Table 1). However, this enhancement was only observed for the combinations of daidzein and genistein with gentamicin, tobramycin, and ampicillin against MRPA (Figure 4). Synergistic or partially synergistic FICI results were observed only for combinations of each of gentamicin, tobramycin, and ampicillin with daidzein or with genistein against MRPA (Table 2). Therefore, based on their synergistic effects with antibiotics against PAO1 and MRPA strains, the combination of daidzein and genistein with gentamicin was selected for subsequent in vivo assay.

**Figure 5:**
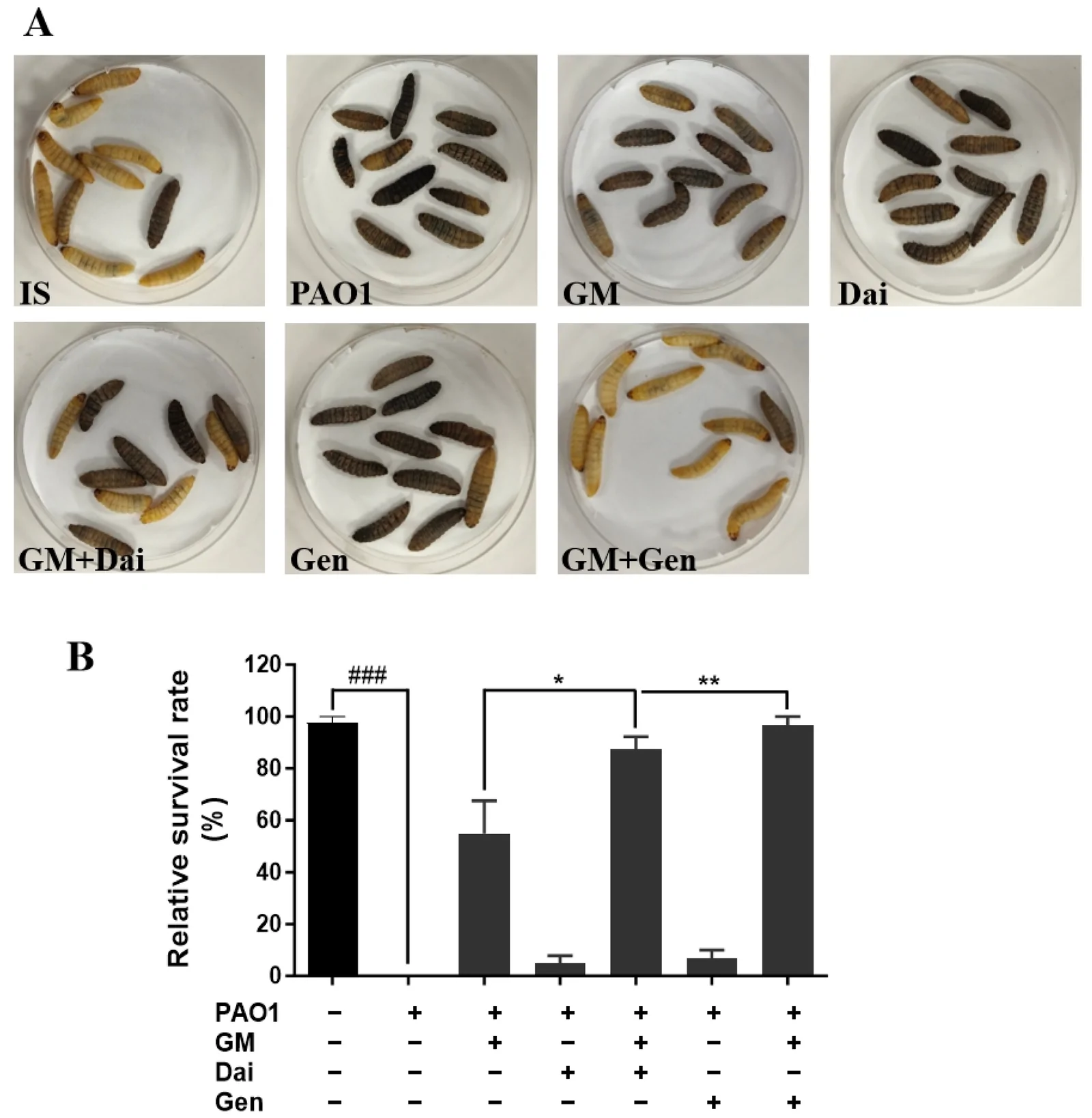
Daidzein and genistein enhance the efficacy of gentamicin against *P. aeruginosa* in a *Galleria mellonella* infection model. (**A**) *G. mellonella* larvae (n = 10 per group) were injected with approximately 1 × 10^6^ CFU/mL of *P. aeruginosa* PAO1 (diluted 1:100 from an overnight culture) that had been pre-treated with gentamicin (GM) alone or in combination with daidzein (Dai) and genistein (Gen) at 1 μM. Saline solution-injected larvae served as the negative control, and larvae injected with bacteria alone (without drug treatment) served as the untreated infection control. After injection, larvae were incubated at 20 °C for 15 h, and larval survival and melanization were observed. (**B**) The survival rates were presented graphically. *, *p* < 0.05 and **, *p* < 0.01; ###, *p* < 0.001.

To confirm the synergistic anti-virulence effects of daidzein and genistein, a *Galleria mellonella* infection model was utilized. As the 1 μM and 100 μM concentrations exhibited comparable antibiotic synergy according to MIC and FICI analysis (Figure 4 and Table 1), the 1 μM dose was chosen for this in vivo infection study. Compared with the blank group (LB medium), diluted *P. aeruginosa* culture (1:100) exhibited strong toxicity regardless of the presence of 1 μM daidzein or genistein (Figure 5). Although gentamicin at its MIC showed anti-toxic activity, it only moderately improved the survival rate and larval melanization of *G. mellonella* (Figure 5). In the *G. mellonella* infection model, recognition of bacterial LPS/LTA triggers the release of prophenoloxidase from oenocytoids, which is subsequently converted to active phenoloxidase (PO) via a serine protease cascade. PO catalyzes the oxidation of phenols to quinones, which polymerize into melanin and result in larval melanization. This melanization cascade contributes to the antimicrobial activity against bacteria, and its degree depends on both bacterial virulence and inoculum size [30]. However, the addition of either daidzein or genistein in combination with gentamicin significantly increased both larval survival and melanization. Notably, the combination of genistein with gentamicin was the most effective (Figure 5). All results suggested that daidzein and genistein were potential adjuncts to antibiotics against *P. aeruginosa*.

### 2.4. Daidzein and Genistein Inhibited the Production of Inflammatory Cytokines When Combined with RXR Agonist

**Figure 6:**
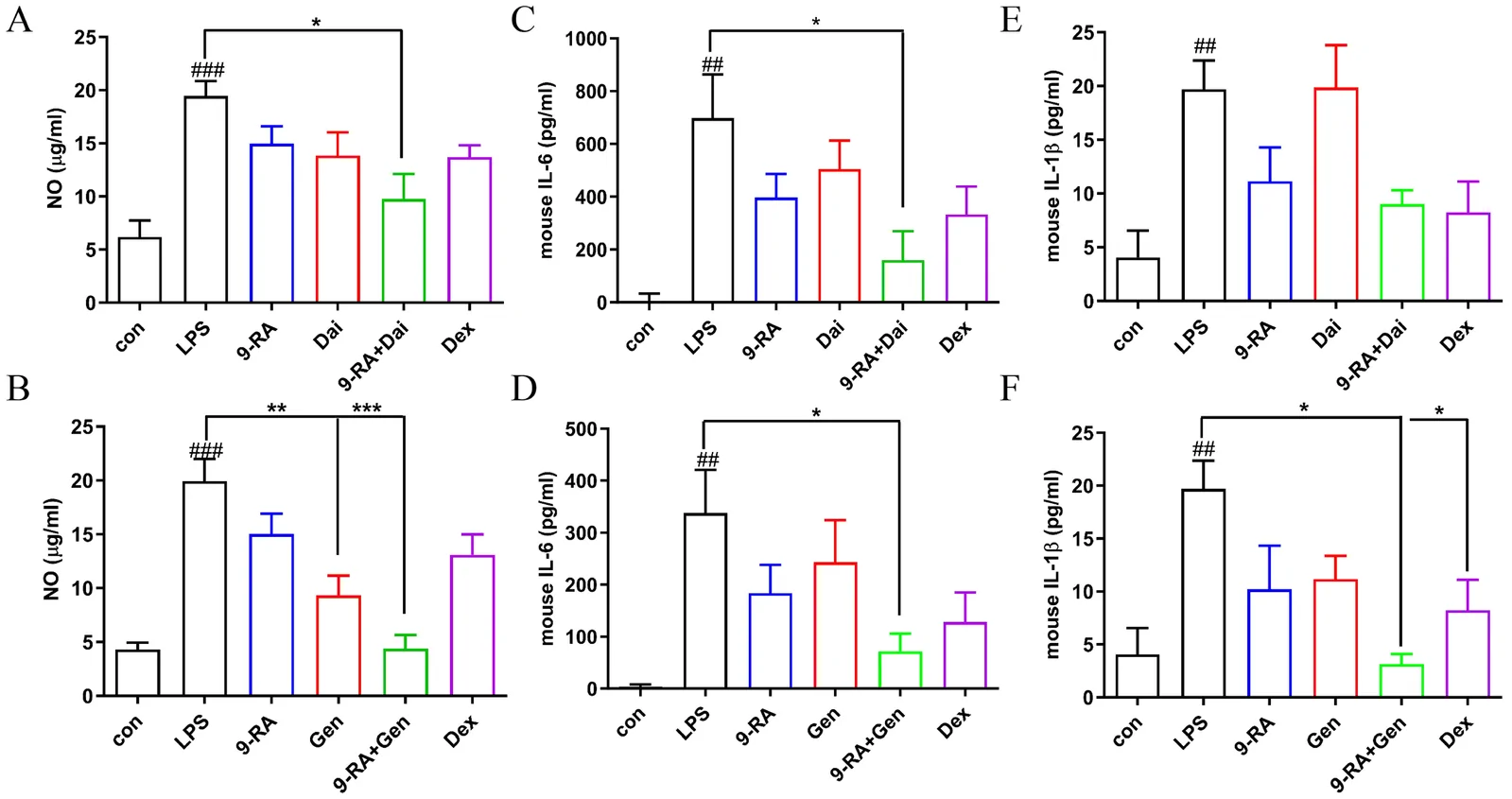
Daidzein and genistein enhance the efficacy of 9-cis-retinoic acid in inhibiting LPS-induced inflammatory responses in RAW264.7 macrophages. (**A**,**B**) Nitric oxide (NO) production was measured in the culture supernatants using the Griess reagent after 24 h of treatment with daidzein (**A**) or genistein (**B**) at 10 μM, alone or in combination with 9-cis-retinoic acid (9-RA, 10 μM), followed by stimulation with 0.1 μg/mL LPS. (**C**–**F**) Production of the pro-inflammatory cytokines interleukin-6 (IL-6) (**C**,**D**) and interleukin-1β (IL-1β) (**E**,**F**) was determined by murine-specific ELISA kits in the culture supernatants after treatment with daidzein (**C**,**D**) or genistein (**E**,**F**) alone or in combination with 9-RA. Dexamethasone (Dex, 10 μM) was employed as the positive control. *, *p* < 0.05; **, *p* < 0.01 and ***, *p* < 0.001; ##, *p* < 0.01, and ###, *p* < 0.001.

As shown in Figure 1, daidzein and genistein were evaluated for their ability to activate PPAR-γ. However, the activity of their combination with the RXR agonist (9-cis-retinoic acid) remains unknown. Therefore, in this study, the anti-inflammatory effects of combining daidzein and genistein with 9-cis-retinoic acid were investigated. Prior to evaluating the anti-inflammatory effects of these two isoflavones, a cell viability assay was performed to test their cytotoxicity. The results demonstrated that daidzein and genistein exhibited no significant cytotoxicity in Raw264.7 cells up to 40 μM (Figure S2). Therefore, 10 μM was used for the NO and ELISA assays. When administered alone, daidzein, genistein, or 9-cis-retinoic acid weakly inhibited LPS-induced NO production and the expression of inflammatory factors interleukin-6 (IL-6) and interleukin-1β (IL-1β) (Figure 6). In contrast, the combination of daidzein with 9-cis-retinoic acid significantly suppressed both NO production and IL-6 expression (Figure 6 and Figure S3). Similarly, the combination of genistein with 9-cis-retinoic acid markedly reduced NO production as well as IL-6 and IL-1β expression (Figure 6D–F). Dexamethasone (Dex, 10 μM) was employed as the positive control. However, none of the compounds exhibited appreciable inhibition of tumor necrosis factor-alpha (TNF-α) at 10 μM (Figure S4). Therefore, all results indicated that the combination of daidzein and genistein with 9-cis-retinoic acid could suppress LPS-induced inflammation through activating the PPAR-γ/RXR signaling pathway.

## 3. Discussion

*P. aeruginosa* is a major health challenge that causes serious acute and chronic infections and presents high morbidity and mortality rates [5,31]. Anti-pathogenic or anti-virulence strategies, which target virulence mechanisms without suppressing bacterial growth, have emerged as an alternative approach that also modulates host inflammatory responses [27,32,33]. By preserving bacterial viability, anti-pathogenic agents minimize selective pressure and the associated risk of resistance. Consequently, they convert pathogens into a functionally non-pathogenic state, preventing disease without driving resistance. Therefore, anti-pathogenic agents might be a potential antibiotic adjuvant.

QS system inhibitors have been proposed as potential anti-pathogenic agents by modulating both bacterial virulence mechanisms and host physiological responses. In our previous study, chermesiterpenoid B (Che B) seco acid methyl ester (Che B ester) and Che B isolated from a marine-derived fungus were found to reduce protease production and decrease the infectivity of *P. aeruginosa* through anti-QS and PPAR-γ activation [22]. However, their application is limited by low natural abundance. In the present study, daidzein and genistein were demonstrated to be potential anti-pathogenic agents based on their optimal anti-QS and PPAR-γ activation activities. Moreover, daidzein and genistein were found to suppress both pyocyanin production and biofilm formation in *P. aeruginosa* without disrupting bacterial growth, suggesting that this strategy exerts minimal selective pressure for the development of resistance. Different from Che B and Che B ester, daidzein and genistein exist not only in marine-derived natural products but also in a variety of fruits, vegetables, and whole grains, particularly in soybeans, which are commonly consumed in daily life. Moreover, they have been proven to be safe and exhibit modest efficacy in estrogenic effects, neurological protection, anti-cancer, anti-osteoporotic, and cardioprotective activities [34]. Significantly, daidzein and genistein are effective and food-derived compounds that warrant further development as next-generation anti-pathogenic agents within the medicine–food homology framework.

Virulence factors such as pyocyanin contribute to biofilm formation and damage host tissues by disrupting calcium balance or promoting neutrophil apoptosis [35]. Furthermore, bacterial biofilms enhance antibiotic resistance and promote persistent host inflammation [35]. Significantly, further experiments confirmed that daidzein and genistein synergistically sensitized *P. aeruginosa* to gentamicin, carbenicillin, tobramycin, ampicillin, and polymyxin B. In addition, they synergistically sensitized multidrug-resistant *P. aeruginosa* to gentamicin and exerted partial synergistic effects against tobramycin and ampicillin. Moreover, these synergistic effects were also confirmed using a *Galleria mellonella* infection model. Collectively, these results demonstrate that daidzein and genistein are potential adjuncts to antibiotics against *P. aeruginosa* both in vitro and in vivo. It should be noted that while our in vitro results demonstrated the synergistic effects of daidzein and genistein with multiple antibiotics, only the gentamicin combination was assessed in vivo. Future studies are warranted to systematically evaluate other antibiotic combinations in different infection models and to explore the underlying molecular mechanisms of these synergistic interactions.

After *P. aeruginosa* infection, the virulence factors and biofilms stimulate host cells and induce acute and chronic inflammation, respectively. Accumulating evidence has proved that both daidzein and genistein inhibit inflammatory responses by activating the PPAR-γ signaling pathway [36,37]. In this study, luciferase and ELISA assays also confirmed that daidzein and genistein inhibited LPS-induced inflammation. However, these effects were weak at 10 μM. Interestingly, RXRs are common heterodimerization partners of PPARs; the combination of PPAR (especially PPAR-γ) agonists with RXR agonists was proved to exert synergistic effects [38], which may enhance their respective anti-inflammatory activities. Nevertheless, the anti-inflammatory activity of daidzein and genistein combined with RXR agonist (9-cis-retinoic acid) has remained unknown. Therefore, in this study, we demonstrate for the first time that daidzein and genistein promote 9-cis-retinoic acid-mediated inhibition of LPS-induced NO, IL-6, and IL-1β production. Taken together, all results demonstrated that daidzein and genistein are effective anti-pathogenic agents, exerting multifaceted (bacteria-directed and host-directed) actions against *P. aeruginosa* infection. However, all cell-based assays were performed in murine RAW264.7 macrophages. While this is a widely used model for inflammation and PPAR-γ signaling, we acknowledge that studies in human cell lines (e.g., THP-1-derived macrophages) would strengthen translational relevance. Future work should address this in human immune cells.

## 4. Materials and Methods

### 4.1. Bacterial Strains, Plasmids and Cell Culture Conditions

All bacterial strains and plasmids were obtained from the Microbiology Laboratory (Professor Lee Joon-Hee) at Pusan National University. The wild-type (PAO1) and multidrug-resistant (MRPA) strains (KNRRB 2200) of *P. aeruginosa* were grown in Luria–Bertani medium (LB; 10 g/L tryptone, 5 g/L yeast extract, and 5 g/L NaCl) and M63-Gly medium (including KH_2_PO_4_, 3 g/L; K_2_HPO_4_, 7 g/L; (NH_4_)_2_SO_4_, 2 g/L; 1 mM MgSO_4_; 0.2% glycerol).

Compounds were dissolved in cell culture-grade DMSO (Solarbio, Beijing, China, ≥99.9%) as 50 mM stocks and stored at −20 °C. Working concentrations were prepared by dilution in media immediately before use. Final DMSO was ≤0.1% (*v*/*v*) for all assays and ≤0.2% (*v*/*v*) for MIC tests, both of which were verified to have no effect on bacterial or mammalian cell viability.

Ac2F (rat liver) and RAW264.7 (mouse macrophage) cells were purchased from the American Type Culture Collection (ATCC, Manassas, VA, USA) and maintained in DMEM containing 10% FBS (Gibco, Waltham, MA, USA), 100 U/mL penicillin and 100 μg/mL streptomycin (Beyotime, Shanghai, China) at 37 °C under 5% CO_2_. Cells were passaged every 2 days at 80–90% confluence and used in the logarithmic growth phase for all experiments.

### 4.2. Measurement of Anti-QS Activity

In order to detect the activity of LasR in the *E. coli* system, two plasmids pJN105L (lasR open reading frame in pJN105, gentamicin resistance) and pSC11 (*lasIp-lacZ* reporter in pQF50, ampicillin and carbenicillin resistance) were cotransformed into DH5α competent cells. Then, this reporter strain was incubated in LB medium, which included 10 μg/mL gentamicin and 100 μg/mL ampicillin at 37 °C in a shaking incubator. When the bacterial cultures reached an OD_600_ of 0.3, 10 μM of daidzein and genistein, 50 nM of 3OC12-HSL, and 0.4% arabinose were added. After culture for 2 h, a β-Galactosidase kit was used to measure the activity of LasR.

To confirm the anti-LasR activity of the compounds against *P. aeruginosa*, a reporter strain was constructed by transforming pSC11 into PAO1 competent cells. The resulting strain was cultured overnight in LB medium containing 10 μg/mL carbenicillin, after which 10 μM of daidzein and genistein were added. The anti-LasR activity was measured by the Galacto-Light Plus kit (Applied Biosystems, Foster City, CA, USA), as described elsewhere [22]. Final β-galactosidase activities were calculated as luminescence/OD_600_ and expressed as relative values to the inhibitor-free control.

### 4.3. Luciferase Reporter Assay

Ac2F cells were seeded into 48-well plates and cultured overnight. When the cells reached 90% confluence, the PPRE-x3-TK-luciferase reporter plasmid and the full-length human PPAR-γ1 expression vector were co-transfected into the cells using DMEM medium without serum or supplements. After transfection, complete DMEM medium (with 10% FBS) was used to culture cells for 15 h. Then, the cells were treated with 10 μM of daidzein, genistein, prunetin, and glycitein using DMEM medium without serum and incubated for 6 h. Rosiglitazone was employed as a positive compound. After drug treatment, the cells were lysed with lysis buffer and measured using the ONE-Glo^TM^ Luciferase Assay System reagent using the GloMax^®^-Multi Microplate Multimode Reader (Promega Co., Madison, WI, USA), as described elsewhere [22].

### 4.4. Minimum Inhibitory Concentration (MIC) Assay

The MIC values of antibiotics alone or in combination with daidzein/genistein were determined using the broth microdilution method in 96-well plates. Bacterial strains were grown overnight in 5 mL of LB at 37 °C. Bacteria from an overnight culture were diluted to OD_600_ = 0.1, then 1000-fold in LB to 2 × 10^4^ CFU/mL. An aliquot (49 µL) was mixed with 1 µL of compound dilutions in 96-well plates, yielding a final inoculum of 1 × 10^3^ CFU/mL per well. Daidzein and genistein were tested at concentrations ranging from 0.08 to 10 mM (two-fold serial dilutions). For antibiotic combination assays, daidzein and genistein were applied at 1 μM and 100 μM. The antibiotic concentration ranges used for MIC determination are presented in Table 3. After incubation at 37 °C for 24 h, the MIC values were determined by visual inspection of bacterial growth.

The synergistic effects of antibiotics combined with daidzein or genistein were evaluated using the checkerboard broth microdilution method in 96-well plates. The fractional inhibitory concentration (FIC) was calculated as follows:

$$FIC\;=\;\frac{MIC\;of\;drug\;A\;or\;B\;in\;combination}{MIC\;of\;drug\;A\;or\;B\;alone}$$

The fractional inhibitory concentration index (FICI) was then calculated as the sum of the individual FICs: FICI = FIC_A + FIC_B. A FICI ≤ 0.5 was considered to indicate a synergistic effect; 0.5 < FICI ≤ 0.75 was partial synergy; 0.75 < FICI ≤ 1 was additive [39].

### 4.5. Biofilm Formation Assay

PAO1 cells were cultured overnight in LB medium at 37 °C with shaking. The overnight culture was diluted 100-fold in M63-Gly medium (approximately 4 × 10^6^ CFU/mL). Then, 1 μM daidzein and genistein were co-cultured with the cells in 96-well plates for 24 h at 37 °C. After drug treatment, the OD_600_ was measured to assess cell density. The cells were then washed with water and stained with 0.1% crystal violet for 10 min. Subsequently, 30% acetic acid was added to solubilize the retained crystal violet, and the absorbance was measured at 600 nm (A_600_) using a microplate reader. Biofilm formation was quantified by A_600_ and normalized to cell density (OD_600_). Data are expressed as relative percentages to the untreated control (set as 100%).

The percentage of biofilm formation was calculated using the following formula:

$$Biofilm\;formation\;(\%)\;=\;\frac{A600\;of\;experimental\;well}{OD600\;of\;experimental\;well}/\frac{A600\;of\;control\;well}{OD600\;of\;control\;well}\;\times \;100\%$$

### 4.6. Pyocyanin Generation Assay

For pyocyanin quantification, the bacterial culture supernatant was collected after centrifugation, filtered through a 0.22 μm filter, and mixed with an equal volume of chloroform. After centrifugation (6000 rpm, 5 min), the lower chloroform layer was collected and mixed with 0.2 M HCl with vortexing until a pale red color developed. Absorbance was measured at 520 nm, which correlates directly with pyocyanin levels.

### 4.7. Virulence Assay of P. aeruginosa

A *Galleria mellonella* infection model [30] was used to evaluate the effect of the daidzein/genistein combined with antibiotics on the virulence of PAO1. PAO1 cells treated with daidzein/genistein (1 μM) and gentamicin (GM; 3.6 μg/mL) were cultured until an OD_600_ of 2.0. The bacterial culture was then diluted 100-fold with insect saline (IS) to approximately 1 × 10^6^ CFU/mL. Subsequently, 10 μL of the diluted bacterial suspension was injected into each *G. mellonella* larva. The larvae were incubated at 20 °C for 15 h, after which larval survival and melanization were monitored. In the control group, larvae were injected with 10 μL of IS alone.

### 4.8. Nitric Oxide (NO) Production

RAW264.7 macrophages were pretreated with the indicated compounds for 1 h, after which they were stimulated with 0.1 μg/mL lipopolysaccharide (LPS) for 24 h. The culture supernatants were then collected, and 80 μL of each supernatant was mixed with 80 μL of Griess reagent in 96-well plates. The mixture was incubated at 37 °C in the dark for 15 min. Absorbance was measured at 520 nm using a microplate reader. A standard curve for nitric oxide (NO) was generated using sodium nitrite at concentrations ranging from 0 to 100 μM. Dexamethasone was employed as a positive control.

### 4.9. ELISA Assay

RAW264.7 cells were pretreated with the test compounds for 1 h, followed by stimulation with 0.1 μg/mL LPS for 24 h. The culture supernatants were then collected, and the levels of interleukin-6 (IL-6), interleukin-1β (IL-1β), and tumor necrosis factor-α (TNF-α) in the supernatants were measured using murine-specific ELISA kits (BioLegend, San Diego, CA, USA) according to the manufacturer’s instructions. Optical density was measured at 450 nm and corrected with a reference wavelength of 570 nm for all three cytokines.

### 4.10. Docking Simulation

The crystal structures of LasR (PDB ID: 4NG2) and PPAR-γ (PDB ID: 2PRG) were downloaded from the Protein Data Bank (https://www.rcsb.org, accessed on 10 June 2026). The proteins were prepared by adding hydrogen atoms, removing water molecules, and performing energy minimization. Daidzein and genistein were prepared by adding hydrogen atoms followed by energy minimization. The binding grids were generated based on the co-crystallized ligands of 4NG2 and 2PRG, and molecular docking was simulated using the LibDock protocol in Discovery Studio software 2019.

### 4.11. Molecular Dynamics (MD) Simulations

Molecular dynamics (MD) simulations were performed using GROMACS to investigate the binding stability of daidzein and genistein with LasR and PPAR-γ, based on the molecular docking results. The CHARMM36m force field was employed for all simulations. Topology and parameter files for each protein–ligand complex were generated using the CHARMM-GUI web server. Each system was solvated in a cubic periodic box with TIP3P water molecules, neutralized with Na^+^/Cl^−^ ions, and subjected to energy minimization using the steepest descent algorithm. Equilibration was conducted under NVT and NPT ensembles. Production MD runs were performed for 100 ns. Trajectory analyses, including root-mean-square deviation (RMSD), root-mean-square fluctuation (RMSF), and radius of gyration (Rg), were conducted to assess conformational stability and residue-level flexibility.

### 4.12. Statistical Analysis

All experiments were performed with at least three independent biological replicates, each with triplicate technical measurements. All results are expressed as mean ± SEM, and statistical comparisons were conducted using one-way ANOVA followed by Tukey’s post hoc test for multiple comparisons (GraphPad Prism 11.1.0, trial version). Significance levels are indicated as * *p* < 0.05, ** *p* < 0.01, and *** *p* < 0.001, with *p* < 0.05 considered statistically significant.

## 5. Conclusions

Daidzein and genistein were selected based on their optimal anti-LasR and PPAR-γ activation activities. They suppressed pyocyanin production and biofilm formation in *P. aeruginosa* without affecting bacterial growth. It is suggested that daidzein and genistein act as QS inhibitors, exhibiting anti-pathogenicity activity with a low likelihood of inducing antibiotic resistance, and may serve as antibiotic sensitizers. Significantly, daidzein and genistein were found to synergistically sensitize PAO1 to gentamicin, carbenicillin, tobramycin, ampicillin, and polymyxin B, and sensitize MRPA to gentamicin, tobramycin, and ampicillin. In addition, a *Galleria mellonella* infection model confirmed that daidzein and genistein significantly enhance the efficacy of gentamicin against *P. aeruginosa* infection in vivo. Furthermore, daidzein and genistein were also found to promote the anti-inflammatory activity of 9-cis-retinoic acid through activating PPAR-γ in host macrophages. In summary, daidzein and genistein may serve as anti-pathogenicity agents that sensitize antibiotics against *P. aeruginosa* and inhibit host inflammatory responses through QS inhibition and PPAR-γ activation.

## Figures and Tables

**Table 1:** The fractional inhibitory concentration index (FICI) of antibiotics combined with daidzein and genistein against *P. aeruginosa* PAO1.

FICI	Daidzein		Genistein	
	1 μM	100 μM	1 μM	100 μM
GM	0.29 ± 0.13	0.33 ± 0.13	0.28 ± 0.08	0.35 ± 0.15
Car	0.38 ± 0.14	0.33 ± 0.13	0.29 ± 0.10	0.35 ± 0.17
Tob	0.29 ± 0.13	0.34 ± 0.12	0.25 ± 0.15	0.32 ± 0.14
Amp	0.29 ± 0.17	0.31 ± 0.15	0.35 ± 0.17	0.33 ± 0.13
Poly B	0.43 ± 0.12	0.43 ± 0.12	0.36 ± 0.14	0.39 ± 0.14

Gentamicin: GM; Carbenicillin: Car; Tobramycin: Tob; Ampicillin: Amp; Polymyxin B: Poly B. FICI ≤ 0.5, synergistic effect; 0.5 < FICI ≤ 0.75, partial synergy; 0.75 < FICI ≤ 1, additive.

**Table 2:** The fractional inhibitory concentration index (FICI) of antibiotics combined with daidzein and genistein against multidrug-resistant *P. aeruginosa* MRPA.

FICI	Daidzein		Genistein	
	1 μM	100 μM	1 μM	100 μM
GM	0.46 ± 0.09	0.46 ± 0.09	0.71 ± 0.27	0.46 ± 0.09
Car	1.10 ± 0.55	1.20 ± 0.76	1.00 ± 0.61	1.20 ± 0.76
Tob	0.75 ± 0.22	0.71 ± 0.19	0.65 ± 0.23	0.67 ± 0.30
Amp	0.58 ± 0.17	0.70 ± 0.18	0.72 ± 0.24	0.65 ± 0.18
Poly B	0.92 ± 0.20	0.92 ± 0.20	0.75 ± 0.27	0.75 ± 0.27

Gentamicin: GM; Carbenicillin: Car; Tobramycin: Tob; Ampicillin: Amp; Polymyxin B: Poly B. FICI ≤ 0.5, synergistic effect; 0.5 < FICI ≤ 0.75, partial synergy; 0.75 < FICI ≤ 1, additive.

**Table 3:** Concentration ranges for MIC assays.

Compounds/Antibiotics	The Concentration Range for MIC Assay (µg/mL)	
	PAO1	MRPA
GM	0.625–80	218.75–14,000
Car	8–1024	8–1024
Tob	0.12–15.2	31.25–2000
Amp	7.8–1000	562.5–9000
Poly B	0.12–15.6	0.4875–62.4

Gentamicin: GM; Carbenicillin: Car; Tobramycin: Tob; Ampicillin: Amp; Polymyxin B: Poly B.

## Data Availability

The raw data supporting the conclusions of this article will be made available by the authors on request.

## Supplementary Materials

The following supporting information can be downloaded at: https://www.mdpi.com/article/10.3390/antibiotics15090829/s1, Figure S1: Molecular docking and molecular dynamics simulation analyses of daidzein and genistein binding to LasR and PPAR-γ; Figure S2: The cell viability of Raw264.7 cells following treatment with daidzein and genistein; Figure S3: NO production in Raw264.7 cells following treatment with daidzein, genistein, and 9-cis-retinoic acid under LPS stimulation; Figure S4: The production of TNF-α was evaluated by ELISA assay after treatment with daidzein and genistein alone or in combination with 9-cis-retinoic acid; Table S1: The Interactions of daidzein and genistein with LasR and PPAR-γ.

## Abbreviations

The following abbreviations are used in this manuscript:

3OC12-HSL	N-(3-oxododecanoyl)-L-homoserine lactone
Amp	ampicillin
Car	carbenicillin
CFU	Colony-Forming Unit
ELISA	Enzyme-Linked Immunosorbent Assay
FIC	Fractional Inhibitory Concentration
FICI	Fractional Inhibitory Concentration Index
GM	Gentamicin
IL-1β	Interleukin-1β
IL-6	Interleukin-6
LPS	Lipopolysaccharide
MIC	Minimum Inhibitory Concentration
NO	Nitric Oxide
Poly B	polymyxin B
PPAR	Peroxisome Proliferator-Activated Receptor
PPAR-γ	Peroxisome Proliferator-Activated Receptor γ
PPRE	Peroxisome Proliferator Response Elements
QS	Quorum Sensing
QSIs	Quorum Sensing Inhibitors
Rg	Radius of gyration
RMSD	Root-Mean-Square Deviation
RMSF	Root-Mean-Square Fluctuation
RXR	Retinoid X Receptor
TNF-α	Tumor Necrosis Factor-α
Tob	tobramycin

## References

[1] Qin S., Xiao W., Zhou C., Pu Q., Deng X., Lan L., Liang H., Song X., Wu M. (2022). *Pseudomonas aeruginosa*: Pathogenesis, virulence factors, antibiotic resistance, interaction with host, technology advances and emerging therapeutics. Signal Transduct. Target. Ther..

[2] Zhang Y., Sass A., Van Acker H., Wille J., Verhasselt B., Van Nieuwerburgh F., Kaever V., Crabbé A., Coenye T. (2018). Coumarin Reduces Virulence and Biofilm Formation in *Pseudomonas aeruginosa* by Affecting Quorum Sensing, Type III Secretion and C-di-GMP Levels. Front. Microbiol..

[3] Chen K., Fu Q., Li D., Wu Y., Sun S., Zhang X. (2016). Wnt3a suppresses *Pseudomonas aeruginosa*-induced inflammation and promotes bacterial killing in macrophages. Mol. Med. Rep..

[4] Blomquist K.C., Nix D.E. (2021). A Critical Evaluation of Newer β-Lactam Antibiotics for Treatment of *Pseudomonas aeruginosa* Infections. Ann. Pharmacother..

[5] Pang Z., Raudonis R., Glick B.R., Lin T.J., Cheng Z. (2019). Antibiotic resistance in *Pseudomonas aeruginosa*: Mechanisms and alternative therapeutic strategies. Biotechnol. Adv..

[6] Xiao G., Li J., Sun Z. (2023). The Combination of Antibiotic and Non-Antibiotic Compounds Improves Antibiotic Efficacy against Multidrug-Resistant Bacteria. Int. J. Mol. Sci..

[7] Cooper M.A., Shlaes D. (2011). Fix the antibiotics pipeline. Nature.

[8] Walsh C. (2000). Molecular mechanisms that confer antibacterial drug resistance. Nature.

[9] Letizia M., Diggle S.P., Whiteley M. (2025). *Pseudomonas aeruginosa*: Ecology, evolution, pathogenesis and antimicrobial susceptibility. Nat. Rev. Microbiol..

[10] Chen J., Wang B., Lu Y., Guo Y., Sun J., Wei B., Zhang H., Wang H. (2019). Quorum Sensing Inhibitors from Marine Microorganisms and Their Synthetic Derivatives. Mar. Drugs.

[11] Kiran S., Sharma P., Harjai K., Capalash N. (2011). Enzymatic quorum quenching increases antibiotic susceptibility of multidrug resistant *Pseudomonas aeruginosa*. Iran. J. Microbiol..

[12] Gupta P., Chhibber S., Harjai K. (2015). Efficacy of purified lactonase and ciprofloxacin in preventing systemic spread of *Pseudomonas aeruginosa* in murine burn wound model. Burns.

[13] Mion S., Rémy B., Plener L., Brégeon F., Chabrière E., Daudé D. (2019). Quorum Quenching Lactonase Strengthens Bacteriophage and Antibiotic Arsenal Against *Pseudomonas aeruginosa* Clinical Isolates. Front. Microbiol..

[14] Shukla A., Shukla G., Parmar P., Patel B., Goswami D., Saraf M. (2021). Exemplifying the next generation of antibiotic susceptibility intensifiers of phytochemicals by LasR-mediated quorum sensing inhibition. Sci. Rep..

[15] Bedi B., Maurice N.M., Ciavatta V.T., Lynn K.S., Yuan Z., Molina S.A., Joo M., Tyor W.R., Goldberg J.B., Koval M. (2017). Peroxisome proliferator-activated receptor-γ agonists attenuate biofilm formation by *Pseudomonas aeruginosa*. FASEB J..

[16] Tyagi S., Gupta P., Saini A.S., Kaushal C., Sharma S. (2011). The peroxisome proliferator-activated receptor: A family of nuclear receptors role in various diseases. J. Adv. Pharm. Technol. Res..

[17] Riquelme S.A., Prince A. (2020). Airway immunometabolites fuel *Pseudomonas aeruginosa* infection. Respir. Res..

[18] Wieland C.W., Siegmund B., Senaldi G., Vasil M.L., Dinarello C.A., Fantuzzi G. (2002). Pulmonary inflammation induced by *Pseudomonas aeruginosa* lipopolysaccharide, phospholipase C, and exotoxin A: Role of interferon regulatory factor 1. Infect. Immun..

[19] Ramirez T., Shrestha A., Kishen A. (2019). Inflammatory potential of monospecies biofilm matrix components. Int. Endod. J..

[20] Zhou J.-P., Yang X.-N., Song Y., Zhou F., Liu J.-J., Hu Y.-Q., Chen L.-G. (2021). Rosiglitazone alleviates lipopolysaccharide-induced inflammation in RAW264.7 cells via inhibition of NF-κB and in a PPARγ-dependent manner. Exp. Ther. Med..

[21] Yu H., Wang L., Huang K., Guo Q., Lin B., Liu Y., Yu M., Ma G. (2022). PPAR-γ agonist pioglitazone alleviates inflammatory response induced by lipopolysaccharides in osteoblast cells. J. Orthop. Res..

[22] Li D., Wang Y., Li H., Niu W., Hong J., Jung J.H., Lee J. (2025). Multifaceted Antipathogenic Activity of Two Novel Natural Products, Chermesiterpenoid B and Chermesiterpenoid B Seco Acid Methyl Ester, Against *Pseudomonas aeruginosa*. Microb. Biotechnol..

[23] Li D., Luo X., Ying W., La Kim E., Hong J., Lee J., Jung J.H. (2023). Peroxisome Proliferator Activated Receptor-γ Agonistic Compounds from the Jellyfish-Derived Fungus *Cladosporium oxysporum*. Chem. Biodivers..

[24] Bruning J.B., Chalmers M.J., Prasad S., Busby S.A., Kamenecka T.M., He Y., Nettles K.W., Griffin P.R. (2007). Partial agonists activate PPARgamma using a helix 12 independent mechanism. Structure.

[25] Garcia-Vallvé S., Guasch L., Tomas-Hernández S., del Bas J.M., Ollendorff V., Arola L., Pujadas G., Mulero M. (2015). Peroxisome Proliferator-Activated Receptor γ (PPARγ) and Ligand Choreography: Newcomers Take the Stage. J. Med. Chem..

[26] Blazer-Yost B.L. (2010). PPARgamma Agonists: Blood Pressure and Edema. PPAR Res..

[27] Dickey S.W., Cheung G.Y.C., Otto M. (2017). Different drugs for bad bugs: Antivirulence strategies in the age of antibiotic resistance. Nat. Rev. Drug Discov..

[28] Simoska O., Sans M., Eberlin L.S., Shear J.B., Stevenson K.J. (2019). Electrochemical monitoring of the impact of polymicrobial infections on *Pseudomonas aeruginosa* and growth dependent medium. Biosens. Bioelectron..

[29] Liu L., Cao X., Ma W., Chen L., Li S., Hu B., Xu Y. (2021). In-situ and continuous monitoring of pyocyanin in the formation process of *Pseudomonas aeruginosa* biofilms by an electrochemical biosensor chip. Sens. Actuators B Chem..

[30] Ménard G., Rouillon A., Cattoir V., Donnio P.-Y. (2021). *Galleria mellonella* as a Suitable Model of Bacterial Infection: Past, Present and Future. Front. Cell. Infect. Microbiol..

[31] Wood S.J., Kuzel T.M., Shafikhani S.H. (2023). *Pseudomonas aeruginosa*: Infections, Animal Modeling, and Therapeutics. Cells.

[32] Ogawara H. (2021). Possible drugs for the treatment of bacterial infections in the future: Anti-virulence drugs. J. Antibiot..

[33] Oh H.-Y., Jalde S.S., Chung I.-Y., Yoo Y.-J., Jang H.-J., Choi H.-K., Cho Y.-H. (2021). An antipathogenic compound that targets the OxyR peroxide sensor in *Pseudomonas aeruginosa*. J. Med. Microbiol..

[34] Intharuksa A., Arunotayanun W., Na Takuathung M., Chaichit S., Prasansuklab A., Chaikhong K., Sirichanchuen B., Chupradit S., Koonrungsesomboon N. (2025). Daidzein and Genistein: Natural Phytoestrogens with Potential Applications in Hormone Replacement Therapy. Int. J. Mol. Sci..

[35] Sawant S., Shaikh S., Baldwin T.C., Karakoula A., Rahman A. (2026). *Pseudomonas aeruginosa*: Associated pathogenesis, epidemiology, resistance mechanisms, host response and emerging treatment strategies. Arch. Microbiol..

[36] Jin S., Zheng Y., Li D., Liu X., Zhu T., Wang S., Liu Z., Liu Y. (2024). Effect of genistein supplementation on microenvironment regulation of breast tumors in obese mice. Breast Cancer Res..

[37] Sakamoto Y., Naka A., Ohara N., Kondo K., Iida K. (2014). Daidzein regulates proinflammatory adipokines thereby improving obesity-related inflammation through PPARγ. Mol. Nutr. Food Res..

[38] Shimizu M., Moriwaki H. (2008). Synergistic Effects of PPARγ Ligands and Retinoids in Cancer Treatment. PPAR Res..

[39] Soltani S., Biron E., Ben Said L., Subirade M., Fliss I. (2022). Bacteriocin-Based Synergetic Consortia: A Promising Strategy to Enhance Antimicrobial Activity and Broaden the Spectrum of Inhibition. Microbiol. Spectr..

