# Supplementary material for: Isoflavones with Multifaceted Activities Synergistically Sensitize Pseudomonas aeruginosa to Antibiotics In Vitro and In Vivo
Source: Antibiotics (Basel). 2026 Aug 26;15(9):829. doi: 10.3390/antibiotics15090829 (PMC13603919; doi:10.3390/antibiotics15090829)
Supplement: Supplementary file 1 [file antibiotics-15-00829-s001.zip › antibiotics-4492979-supplementary.pdf]

antibiotics

Isoflavones with Multifaceted Activities Synergistically Sensitize *Pseudomonas aeruginosa* to Antibiotics In Vitro and In Vivo

Dan-Dan Li [1,†], Tong Xia [1,†], Xin-Yu Zhang [1], Huiyan Li [2], Wen-Xin Niu [2], Tie Yao [3], Joon-Hee Lee [2,4,*] and Li-Li Wang [1,*]

1 School of Chinese Materia Medica, Tianjin University of Traditional Chinese Medicine, Tianjin 301617, China; ldd0930@tjutcm.edu.cn (D.-D.L.); xt15055969110@163.com (T.X.); 13821131526@163.com (X.-Y.Z.)
2 College of Pharmacy, Pusan National University, Busan 46241, Republic of Korea; lihy0522@naver.com (H.L.); niuwenxin@naver.com (W.-X.N.)
3 School of Pharmaceutical Sciences, Liaoning University, Shenyang 110036, China; tieyao1215@163.com
4 Research Institute for Drug Development, Pusan National University, Busan 46241, Republic of Korea
* Correspondence: joonhee@pusan.ac.kr (J.-H.L.); wll1980@126.com (L.-L.W.); Tel.: +82-051-510-2821 (J.-H.L.); +86-022-5959-6238 (L.-L.W.)
† These authors contributed equally to this work.

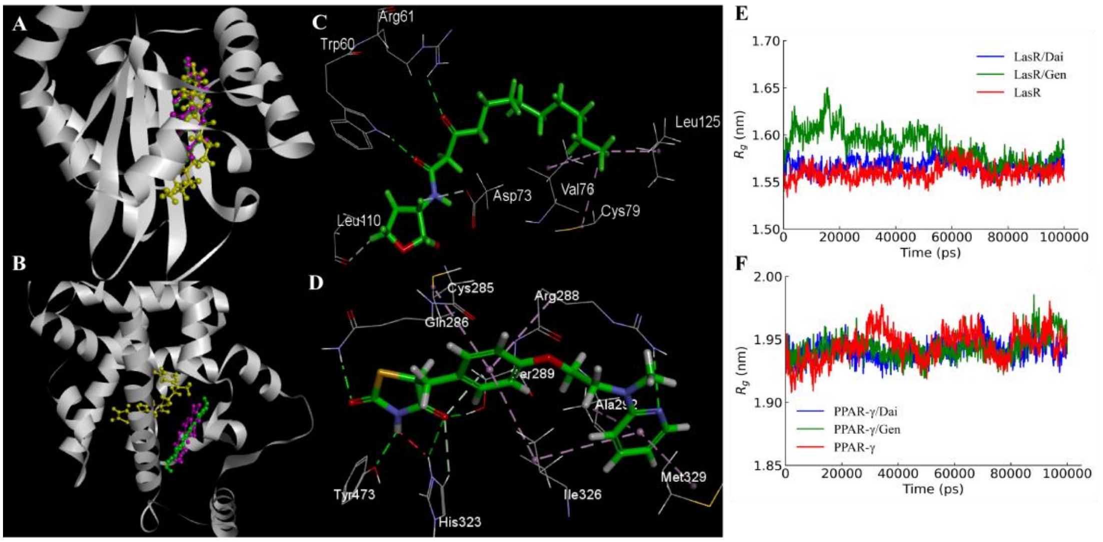

Figure S1. Molecular docking and molecular dynamics simulation analyses of daidzein and genistein binding to LasR and PPAR-γ. Daidzein and genistein are docked into ligand-binding pockets of LasR (A) and PPAR-γ (B). The structure of daidzein is shown in green, and genistein is shown in pink; the standard ligands 3OC12 (A) and rosiglitazone (B) are shown in yellow. (C-D) The binding interactions of 3OC12 and rosiglitazone with LasR and PPAR-γ, respectively. Radius of gyration (Rg) profiles of the LasR (E) and PPAR-γ (F) systems throughout the 100 ns simulation.

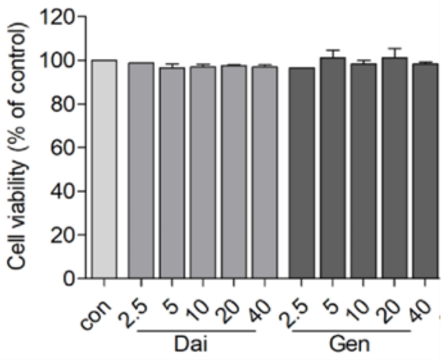

Figure S2. The cell viability of Raw264.7 cells following treatment with daidzein and genistein.

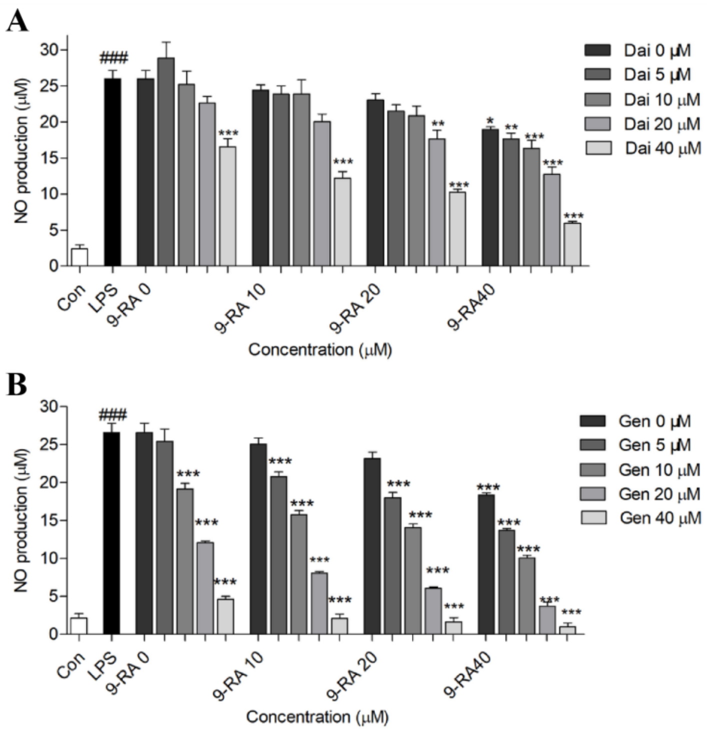

Figure S3. NO production in Raw264.7 cells following treatment with daidzein, genistein, and 9-cis-retinoic acid under LPS stimulation.

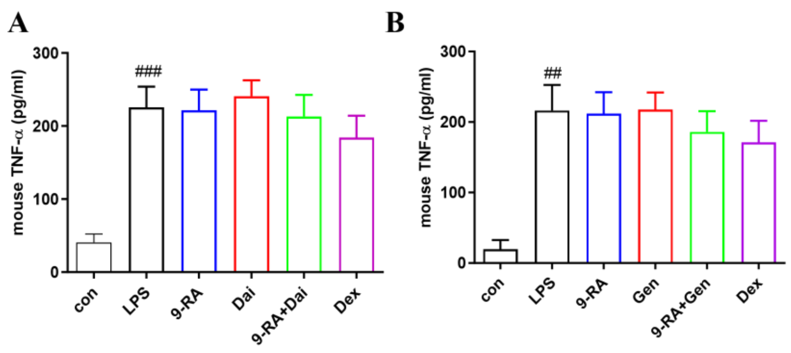

Figure S4. The production of TNF-α was evaluated by ELISA assay after treatment with daidzein and genistein alone or in combination with 9-cis-retinoic acid. Dexamethasone (Dex) was employed as positive drugs. $^{\#\#}$, $p < 0.01$ and $^{\#\#\#}$, $p < 0.001$.

Table S1. The Interactions of daidzein and genistein with LasR and PPAR-γ.

Interactions		LibDockScore	H-Bond	Hydrophobic bond
LasR (4NG2)	Ligand (3OC12-HSL)	137.181	Trp^{60}, Arg^{61}, Asp^{73}, Leu^{110}	Val^{76}, Cys^{79}, Leu^{125}
	Daidzein	118.215	Tyr^{93}, Thr^{115}, Leu^{110}	Leu^{110}, Ala^{105}, Phe^{101}, Tyr^{56}, Leu^{36}, Ala^{127}, Tyr^{64}, Trp^{88}
	Genistein	125.850	Tyr^{93}, Thr^{115}, Leu^{110}, Thr^{75}, Ser^{129}	Leu^{110}, Ala^{105}, Phe^{101}, Tyr^{56}, Leu^{36}, Ala^{127}, Trp^{88}, Tyr^{64}
PPAR-γ (2PRG)	Ligand (rosig-litazone)	146.698	Tyr^{473}, His^{323}, Ser^{289}, Gln^{286}, Arg^{288}	Ile^{326}, Met^{329}, Ala^{292}, Arg^{288}, Cys^{285}
	Daidzein	91.483	Ser^{342}, Ile^{341}, Glu^{343}, Arg^{288}, Glu^{291}, Gly^{284}	Arg^{288}, Leu^{228}
	Genistein	96.438	Ser^{342}	Ile^{341}, Arg^{288}, Cys^{285}, Gly^{284}
